# Prevalence of *Salmonella* Typhimurium and *Salmonella* Enteritidis isolated from poultry meat: virulence and antimicrobial-resistant genes

**DOI:** 10.1186/s12866-023-02908-8

**Published:** 2023-06-15

**Authors:** Marziye Nazari Moghadam, Ebrahim Rahimi, Amir Shakerian, Hassan Momtaz

**Affiliations:** 1 Research Center of Nutrition and Organic Products, Shahrekord Branch, Islamic Azad University, Shahrekord, Iran; 2 Department of Microbiology, Faculty of Basic Sciences, Shahrekord Branch, Islamic Azad University, Shahrekord, Iran

**Keywords:** *Salmonella typhimurium*, Anti-bacterial agents, Serogroup, Trimethoprim, Sulfamethoxazole Drug Combination, Poultry

## Abstract

Salmonellosis, a zoonotic disease, is one of the leading causes of foodborne illness worldwide. It is responsible for most infections caused by consumption of contaminated food. In recent years, a significant increase in the resistance of these bacteria to common antibiotics has been observed, posing a serious threat to global public health. The aim of this study was to investigate the prevalence of virulent antibiotic-resistant Salmonella spp. strains in Iranian poultry markets. A total of 440 chicken meat samples were randomly selected from meat supply and distribution facilities in Shahrekord and tested for bacteriological contamination. After culturing and isolating the strains, identification was performed using the classical bacteriological method and PCR. To determine antibiotic resistance, a disc diffusion test was performed according to the recommendations of the French Society of Microbiology. PCR was used to detect resistance and virulence genes. Only 9% of the samples were positive for Salmonella. These were *Salmonella* typhimurium isolates. All *Salmonella* typhimurium serotypes tested positive for the rfbJ, fljB, invA and fliC genes. Resistance to TET, cotrimoxazole, NA, NIT, piperacillin/tazobactam and other antibiotics was found in 26 (72.2%), 24 (66.7%), 22 (61.1%) and 21 (58.3%) isolates, respectively. The sul1, sul2 and sul3 genes were present in 20, 12 and 4 of 24 cotrimoxazole-resistant bacteria, respectively. Chloramphenicol resistance was found in six isolates, but more isolates tested positive for the floR and cat two genes. In contrast, 2 (33%) of the cat three genes, 3 (50%) of the cmlA genes and 2 (34%) of the cmlB genes were all positive. The results of this investigation showed that Salmonella typhimurium is the most common serotype of the bacterium. This means that most of the antibiotics commonly used in the livestock and poultry industries are ineffective against most Salmonella isolates, which is important for public health.

## Introduction

An important foodborne pathogen known as non-typhoidal *Salmonella* has been linked to the intestinal system of animals that produce food [1]. *Salmonella* is a common foodborne disease associated with the intestinal system of food-producing animals. It is a gram-negative, facultatively anaerobic, flagellated bacteria [2]. Non-typhoidal *Salmonella* causes approximately 153 million cases of gastroenteritis and 57,000 deaths annually worldwide. *Typhimurium* is one of the most common *Salmonella* serovars [3]. Humans and animals are often colonized by *Salmonella* microorganisms, which can proliferate in the intestinal tract and result in a range of gastrointestinal disorders. In some cases, these infections can lead to severe illness or even mortality [4]. Enteritidis, one of the most important *Salmonella* serovars, is a major cause of human illness with symptoms commonly including fever, vomiting, diarrhoea, and abdominal cramps 12–72 h after ingestion of the bacterium [5].

The most prevalent *Salmonella* serotypes causing human gastroenteritis worldwide are *Salmonella Typhimurium* and *Salmonella Enteritidis* [6]. The virulence of *Salmonella* depends on many factors. It is determined by its ability to adhere to cells, to invade them, to survive and to multiply inside epithelial cells and macrophages thanks to the involvement of several virulence factors often carried by plasmids [7]. A significant proportion of these virulence genes are clustered together in specific genomic regions known as “Islands of pathogenicity of *Salmonella*” (SPIs), acquired by horizontal transfer. In general, different virulence factors that are important for a variety of pathogenic mechanisms, including adhesion, invasion, intracellular survival, systemic infection, toxin generation, and iron acquisition, determine how pathogenic *Salmonella* is [8]. An essential feature of the pathogenesis of *Salmonella* is its ability to enter host cells and remain there as an optional intracellular parasite. Another possible risk to human health is the existence of virulence genes that are frequently carried by plasmids, prophages, and *Salmonella* pathogenicity islands (SPIs), which can be transmitted between these bacteria. Several severe *Salmonella* infections associated with these virulent components have been reported [8, 9].

A common source is poultry, and in recent years, much attention has been paid to understanding out how widespread *Salmonella* is at various points in the poultry production chain [10]. In addition, contamination of meat can occur during loading, unloading, and storage. To ensure both meat quality and consumer protection, strict good hygiene and moral slaughter methods must be combined coupled with risk-based preventive actions [11]. The prevalence of drug-resistant strains, which pose serious risks to the public’s health, is another epidemiological issue [12]. Antibiotic resistance has increased, and genes conferring antimicrobial resistance to *Salmonella* are now present because of the selective pressure exerted using antibiotics in poultry production and veterinary medicine for growth promotion and prevention [13–15].

In Iran, numerous studies have been conducted on the incidence of *Salmonella* resistance in humans [16, 17], the factors associated with this infection [18], salmonellosis in eggs [19] and in cattle [20], but no study has considered the virulence and resistance of *Salmonella enteritidis* and *Salmonella typhimurium* strains in Iran. Therefore, this study was carried out to investigate the frequency of virulent strains of *Salmonella enteritidis* and *Salmonella typhimurium* that are resistant to antibiotics in the chicken meat markets of Iran. This study was conducted to determine the frequency of *Salmonella* in poultry meat shops in Iran. The antibiotic resistance patterns of the *Salmonella* isolates and the location of their virulence genes were also identified.

## Methods

### Study framework and sampling

This study was conducted in the city of Shahrekord. The sampling area was selected based on the availability of meat, and shops were randomly selected, along with poultry meat taken tissue samples from each store. The meat sampled was factory farmed. A total of 80 chicken, 80 quail, 80 from grocery stores in Quebec, 80 turkeys, 60 duck and 60 goose were randomly sampled from stores. All samples were collected aseptically, placed in sterile bags and delivered to the Food Microbiology Laboratory of Islamic Azad University of Shahrekord.

### Microbiological analysis

The samples were examined according to the procedure described by Mir-Hassan Moosavy et al. in 2015. Using a sterile scalpel, each tissue sample was cut into pieces weighing approximately 25 g. The pieces were then placed in a sterile mortar and pestle where they were homogenised with 225 ml of buffered peptone water (BPW) before being placed in a 250 ml tube and incubated at 37 °C for 24 to 48 h. The swab samples were then grown on Salmonella-Shigella solid agar medium (containing proteins, lactose and iron) and incubated at 37 °C for 24 h. The suspected *Salmonella* colonies were then examined. In the case of a negative result (*Salmonella* that has lost all colour by this time), the incubation process was extended for a further 24 h. Colonies that were light in colour or had a grey or black centre were considered negative. Questionable colonies on the Salmonella-Shigella agar medium were removed and complementary assays and differentiating microbiological tests were performed, such as urease and IMViC [methyl red (MR) and Voges-Proskauer (VP) broth] [21].

### Detection of virulence genes and ***Salmonella***’s detection

*Salmonella* isolates were grown on Luria Bertani (LB) agar plates and incubated at 37 °C for 24 h prior to DNA extraction. Following to the procedure of Mir-Hassan Moosavy et al. 2015, one loop of each LB agar sample was suspended in 250 µl of sterile distilled water for DNA extraction. Samples were vortexed, boiled for 10 min, and then centrifuged at 6000 x g for 7 min to achieve uniform turbidity. DNA-containing supernatants were collected and stored for multiplex PCR analysis. For DNA amplification of two serovars, *Typhimurium* and *Enteritidis*, multiplex PCR was performed out [16] with two independent sets as previously described. In the case of *Salmonella typhimurium* (Table 1) [20], four sets of primer pairs specific for *rfbJ* (663 bp), *fljB* (526 bp), *invA* (284 bp) and *fliC* (183 bp), and three sets of primer pairs designed for a random sequence specific to the *Salmonella enteritidis* genus *Salmonella* (429 bp), *sefA* (310 bp) and *spv* (250 bp) [22]. Agar gel electrophoresis was used to verify the amplification results. For *Salmonella typhimurium* and *Salmonella enteritidis*, the amplification products were electrophoresed on 1.2% and 1.8% agarose gels, respectively. A 100 bp ladder was used as a molecular weight marker in both methods. When UV was used in the gel documentation system, the gels were stained with ethidium bromide (2 g/mL) to visualise fluorescent bands (BIORAD). For each PCR reaction, positive controls were performed using the reference strains.

**Table 1:** Primers used for the detection of *Salmonella typhimurium* [15]

Primer	Target gene	Length	Sequence (5 ׳ -3 ׳)	Amplification product (bp)
ST139-s	*inv*A	26	GTGAAATTATCGCCACGTTCGGGCAA	284
ST141-as	*inv*A	22	TCATCGCACCGTCAAAGGAACC	284
Rfbj-s	*rfb*J	24	CCAGCACCAGTTCCAACTTGATAC	663
Rfbj-as	*rfb*J	24	GGCTTCCGGCTTTATTGGTAAGCA	663
Flic-s	*fli*C	23	ATAGCCATCTTACCAGTTCCCCC	183
Flic-as	*fli*C	24	GCTGCAACTGTTACAGGATATGCC	183
Fljb-s	*FljB*	24	ACGAATGGTACGGCTTCTGTAACC	526
Fljb-as	*FljB*	24	TACCGTCGATAGTAACGACTTCGG	526

### Antimicrobial susceptibility testing (AST)

The disc diffusion method was used to perform the AST [24, 25]. Incubation was performed at 37 °C for 16–18 h. After incubation, the zones of inhibition by each antibiotic were measured. The reference strain *Salmonella* Typhimurium ATCC 14,028 was used as an internal quality control for AST. The data were interpreted according to the recommendations of the Clinical and Laboratory Standards Institute (CLSI) (CLSI 2018) [24]. Ampicillin (AMP, 20 lg), cefotaxime (CTX, 300 lg), chloramphenicol (C, 30 lg), tetracycline (TET, 30 lg), ciprofloxacin (CIP, 5 lg), gentamicin (GEN, 10 lg), nalidixic acid (NA, 30 lg), cotrimazole (COT, 25 lg), tetracycline (TET, 30 lg), ciprofloxacin (CIP, 5 lg), gentamicin (GEN, 10 lg), nalidixic acid (NA, 30 lg), cotrimazole (COT, 25 lg), tetracycline (TET), nitrofurantion (NIT, 30 lg), imepenem (IPM, 10 lg), meropanem (MRP, 10 lg), piperacillin/tazobactin (PIT, 100/10).

### Antimicrobial resistance gene detection

Target genes conferring resistance to tetracyclines (*tetA, tetB*), sulfonamides (*sul1, sul2*, and *sul3*), chloramphenicol (*cat1, cat2*, and *cat3, cmlA, cmlB, floR*), and aminoglycosides (*aph(3)11a, aac(3)11a*, and *aac6*) were screened by PCR using the corresponding primers in order to The cycle parameters and primer combinations followed the guidelines of Ma et al. (2017) (Table 2) [27]. PCR was performed using 2.0 l of template DNA and 30 l containers containing 3 l of buffer (100 mmol/L Tris-HCl [pH 9], 1.5 mmol/L MgCl2, 500 mmol/L KCl, 0.1% gelatin), 100 lmol/L concentrations of dATP, dTTP, dGTP, and dCTP, 10 pmol of each primer, and 0.9 A thermal cycler (MJ Research, Bio-Rad, Hercules, CA) was used to perform the reactions. Table 3 summarises the cycling conditions and primer sequences [27].

**Table 2:** Primer sequences and their annealing temperatures used in this study (Ma et al. 2007)

Resistance Gene	Primer	Nucleotide sequence 50–30	Product size (bp)	Annealing temperature (°C)	Code of antibiotics
*tet*A	F R	TTGGCATTCTGCATTCACTC GTATAGCTTGCCGGAAGTCG	494	55	TET
*tetB*	F R	CAGTGCTGTTGTGTCATTAA GCTTGGAATACTGAGTGTAA	571	55	TET
*tetC*	F R	CTTGAGAGCCTTCAACCCAG ATGGTCGTCATCTACCTGCC	418	55	TET
*tetD*	F R	GCTCGGTGGTATCTCTGCTC AGCAACAGAATCGGGAACAC	546	55	TET
*tetE*	F R	TATTAACGGGCTGGCATTTC AGCTGTCAGGTGGGTCAAAC	544	55	TET
*tetG*	F R	GCTCGGTGGTATCTCTGCTC CAAAGCCCCTTGCTTGTTAC	550	55	TET
*sul1*	F R	TTTCCTGACCCTGCGCTCTAT GTGCGGACGTAGTCAGCGCCA	793	55	COT
*sul2*	F R	CCTGTTTCGTCCGACACAGA GAAGCGCAGCCGCAATTCAT	667	55	COT
*sul3*	F R	ATGAGCAAGATTTTTGGAATCGTAA CTAACCTAGGGCTTTGGTATTT	792	55	COT
cat1	F R	AACCAGACCGTTCAGCTGGAT CCTGCCACTCATCGCAGTAC	549	55	CHL
cat2	F R	AACGGCATGAACCTGAA ATCCCAATGGCATCGTAAAG	547	55	CHL
cat3	F R	ATCGGCATCGGTTACCATGT ATCCCCTTCTTGCTGATATT	310	55	CHL
cmlA	F R	GGCCTCGCTCTTACGTCATC GCGACACCAATACCCACTAGC	662	55	CHL
cmlB	F R	ACTCGGCATGGACATGTACT ACGGACTGCGGAATCCATAG	840	55	CHL
floR	F R	ATGACCACCACACGCCCCG AGACGACTGGCGACTTCTTCG	198	55	CHL
aac(3)11a	F R	CGGCCTGCTGAATCAGTTTC AAAGCCCACGACACCTTCTC	439	55	GEN
aph(3)11a	F R	TCTGAAACATGGCAAAGGTAG AGCCGTTTCTGTAATGAAGGA	582	55	GEN
aac6	F R	TTGGACGCTGAGATATATGA GCTCCTTTTCCAGAATACTT	476	55	GEN
blaTEM-1	F R	CAGCGGTAAGATCCTTGAGA ACTCCCCGTCGTGTAGATAA	643	55	Control
16 S rDNA	F R	AGAGTTTGATCMTGGCTCAG CCGTCAATTCMTTTRAGTTT	907	55	Control

TET, tetracycline; GEN, gentamicin; COT, cotrimazole

**Table 3:** Prevalence of *Salmonella* spp. in meats

Sample type	No. of samples tested	No. of positive samples	% Prevalence
chicken meat	80	7	8.75
Quail meat	80	6	7.5
Quebec meat	80	9	11.25
Turkey meat	80	7	8.75
Duck meat	60	4	6.75
Goose meat	60	3	5
Overall	400	36	9

### Statistical analysis

For analysis, the data were transferred to a Microsoft Excel spreadsheet (Microsoft Corp., Redmond, Washington). Statistical tests were performed using SPSS (Statistical Package for the Social Sciences) 18.0 (SPSS Inc., Chicago, IL, USA). P values below 0.05 were considered significant.

## Results

### ***Salmonella*** prevalence

*Salmonella-Shigella* solid agar medium was used and differential microbiological tests were conducted, such as urease and IMViC [methyl red broth (MR) and Voges-Proskauer (VP) to isolate and identify *Salmonella*. Out of the 400 samples analyzed, 36 were found to be positive for the culture of *Salmonella*, indicating a contamination rate of 9% (36/400). The prevalence of *Salmonella* in Quebec meat samples (11.25%, 9/80) was significantly higher than in other poultry (Table 3).

### ***Salmonella*** serovars’s detection

The detection of Salmonella serovars was carried out through multiplex PCR using four sets of primer pairs specific for *rfbJ, fljB, invA*, and *fliC* in the case of *Salmonella typhimurium* (Table 1) [23] and three sets of primer pairs designed for a 429 bp fragment specific to the genus *Salmonella enteritidis*, *sefA*, and *spv.* After PCR amplification, all isolates were identified as positive for the *invA* gene. *Salmonella typhimurium* was found to be present in all isolates. The *RfbJ, fljB, invA* and *fliC* genes were observed in all typhimurium serotypes (Table 4).

**Table 4:** Distribution of antimicrobial virulence genes in *Salmonella typhimurium* isolates

Isolates	*invA*	*rfbJ*	*fljB*	*fliC*
chicken meat (n = 80)	7	5	7	6
Quail meat (n = 80)	6	2	5	6
Quebec meat (n = 80)	9	5	8	9
Turkey meat (n = 80)	7	4	7	5
Duck meat (n = 60)	4	-	3	4
Goose meat (n = 60)	3	1	3	2
All *S. Typhimurium* isolates (*n* =36)	36	17	33	35

### Antibiotic sensitivity

The antibiotic sensitivity of each strain was determined by the disk diffusion method. The measured zones of inhibition were interpreted according to the Clinical and Laboratory Standards Institute recommendations. Of the isolates tested, 26 (72.2%), 24 (66.7%), 22 (61.1%), 21 (58.3%), and 21 (58.3%) isolates were resistant to TET, cotrimoxazole, NA, NIT, and piperacillin/tazobactin, respectively. Moreover, resistance to CTX, AMP, GEN, and chloramphenicol was detected in 10–20% of the isolates. A majority (50.5%) of the isolates were at least partially antibiotic resistant (Table 5).

**Table 5:** Antimicrobial resistance profiles of isolated *Salmonella typhimurium*

Antimicrobial resistance	Resistant %	Intermediate %	Susceptible %
TET	26(72.2)	9(25)	1(2.8)
AMP	6(16.7)	10(27.7)	20(55.6)
COT	24(66.7)	11(30.5)	1(2.7)
C	6(16.7)	4(11.2)	26(72.2)
GEN	4(11.2)	2(5.6)	30(83.4)
NA	22(61.2)	8(22.3)	6(16.7)
NIT	21 (58.3%)	11(30.5)	4(11.2)
MRP	5(13.4)	2(5.6)	29(80.6)
CTX	4(11.2)	6(16.7)	26(72.2)
CIP	7(19.5)	2(5.6)	27(75)
IPM	6(16.7)	2(5.6)	28(77.8)
PIT	21 (58.3%)	9(25)	6(16.7)

TET tetracycline, AMP Ampicillin, COT cotrimazole, C Chloramphenicol, GEN gentamicin, NA nalidixic acid, NIT nitrofurantion, MRP meropanem, CTX Cefotaxime, CIP Ciprofloxacin, IPM Imipenem, and PIT piperacilin/tazobactin

The percentage of the total number of isolates resistant, intermediate, or susceptible to a particular antimicrobial is indicated in the last three rows below each antimicrobial

### Detection of resistance genes

To detect the resistance genes to the antibiotics used, a screening of target genes was conducted using PCR. Target genes conferring resistance to tetracyclines (*tetA, tetB*), sulfonamides (*sul1, sul2 and sul3*), chloramphenicol (*cat1, cat2 and cat3, cmlA, cmlB, floR*) and aminoglycosides *(aph(3)11a, aac( 3)11a* and *aac6)* were used. After amplification, the *tetA* gene was present in all 36 TET-resistant isolates, and the *tetB, tetC*, and *tetG* genes were present in 23% (6), 27% (7), and 39% (10) of the isolates, respectively. Additionally, 20 (84%), 12 (50%), and 4 (17%) of the 24 cotrimoxazole-resistant bacteria were detected to possess the sul1, sul2, and sul3 genes, respectively (Table 6). Six isolates were resistant to chloramphenicol, although more isolates were positive for the *floR* and cat two genes, while only two isolates (33%) were positive for the cat three genes, three (50%) were positive for the *cmlA* gene, and two isolates (34%) were positive for the *cmlB* gene.

**Table 6:** Distribution of antimicrobial resistance genes in *Salmonella typhimurium* isolates

isolates	*TetA*	*tetB*	*tetC*	*tetG*	*tetD*	*tetE*	*floR*	*Cat2*	*Cat3*	*Sul 3*	*Sul 1*	*Sul 2*	*cmlA*	*cmlB*
Chicken meat (n = 80)	7	2	2	2	2	1	2	2	1	2	6	5	2	1
Quail meat (n = 80)	6	1	1	1	1	-	-	-	-	-	3	2	-	-
Quebec meat (n = 80)	9	2	2	3	2	-	2	1	1	1	5	4	-	1
Turkey meat (n = 80)	7	1	2	2	-	1	2	2	-	1	4	1	1	-
Duck meat (n = 60)	4	-	-	1	-	-	-	-	-	-	2	-	-	-
Goose meat (n = 60)	3	-	-	1	-	-	-	-	-	-	2	-	-	-
All *S. Typhimurium is* isolates (*n* =400)	36	6	7	10	5	2	6	6	2	4	20	12	3	2

## Discussion

*Salmonella Typhimurium* and *Salmonella Enteritidis* are common causes of foodborne illness and death. With increasing antibiotic resistance, these pathogens have become more prevalent over the years, posing a serious public health problem. The objective of this study was to investigate the frequency of antibiotic-resistant virulent strains of *Salmonella Enteritidis* and *Salmonella Typhimurium* in chicken meat markets in Iran.

In this study, 400 samples were used, among which 36 (9%) samples tested positive for *Salmonella*. This level of contamination is similar to what has been observed in previous investigations in Iran and other countries on various types of meat. A range of Salmonella prevalence has been reported in Iran previously. In this study, compared to the figures reported in other Asian countries such as India (14.8%) [28] and Vietnam (48.9%) [29], China (52.2% [30], Malaysia (48%) [31], Pakistan (38%) [32], and Singapore (18.1%) [33], the prevalence of *Salmonella* in chicken meat was less than 9%. After detecting serovars, we found 36 (9%) positive samples for *S. Typhimurium*. In a similar study, 36 (9%) positive samples for *S. Typhimurium* were reported [34]. In a related survey, *Salmonella Enteritidis, Salmonella Typhimurium*, and *Salmonella Thompson* were the three *Salmonella* serotypes identified by Kumar et al., 2019 [28].

The AST conducted in this study showed that 72.2%, 66.7%, and 61.2% of the identified Salmonella isolates had significant levels of resistance to tetracycline (TET), COT, and NA, respectively. A comparative study conducted in Ibadan, Nigeria, reported a high frequency of isolates resistant to TET (93%), NA (81%), and sulfamethoxazole (87%). However, human isolates showed resistance to trimethoprim, sulfamethoxazole, AMP, and chloramphenicol ranging from 36 to 59%. The diversity of high levels of drug resistance in different locations could be explained by the numerous discoveries on Salmonella antibiotic resistance in various countries. According to Zhao et al. (2017), Salmonella obtained from chickens had considerable resistance to tetracycline (93.1%), but resistance to ampicillin was only 47% [35]. Clinical isolates from the northern region of Nigeria were reported to have 100% resistance to fluoroquinolones in another survey. Kumar (2019) identified high resistance to tetracycline (100%), erythromycin (100%), nalidixic acid (98.57%), ampicillin (95.71%), and ciprofloxacin (82.86%) [28]. Particularly in developing countries, resistance to ciprofloxacin and higher-generation cephalosporins is a serious problem, and chicken-based products may be directly responsible for its establishment [29, 30].

Studies in several parts of Iran show the introduction and growth of antibiotic resistance in Salmonella isolates. Tetracycline (66.6%), furazolidone (52.8%), nalidixic acid (43.8%), lincomycin-spectinomycin (42.3%), flumequine (40.6%), and streptomycin (39.1%) had the highest rates of resistance, according to Peighambari et al. (2011), while ciprofloxacin and imipenem had a sensitivity of 100% [38]. The highest rates of drug resistance were reported during the use of nitrofurantoin (92.6%), nalidixic acid (86.7%), cloxacillin (64%), tetracycline (54%), furazolidone (49.3%), and amoxicillin (45.3%), according to Ezzatpanah et al. (2013) [39]. All isolates were found to be resistant to tetracycline, streptomycin, nalidixic acid, cefazolin, and sulfamethoxazole + trimethoprim by Asadpour et al. (2013) [40]. Drugs with the highest rates of resistance were streptomycin and nalidixic acid (100%), tetracycline (92.3%), neomycin and furazolidone (84.6%), and chloramphenicol (73.3%), according to Raeisi and Ghiyami (2015). Furthermore, amoxicillin and ampicillin (11.5%), ciprofloxacin (7.7%), and gentamicin (3.7%) had the lowest rates of resistance [41]. Tetracycline was first introduced as the most effective antibiotic against *Salmonella* in poultry by Sodagari et al. (2015) in the Alborz province [42]. The need to improve coordination between the veterinary and public health sectors on appropriate diagnosis and notification of foodborne zoonotic pathogens is reinforced by the high level of resistance to NA 22 (61.2%) that was observed in this study, especially in food animals [43]. The result of this study is also highly significant as the effective management of severe or invasive human salmonellosis depends on the use of antibiotics.

In this study, TET-resistant genes were most frequently found in isolates with tetA gene, followed by tetB, tetC, and tetG genes in 23%, 27%, and 39% of TET-resistant isolates, respectively. The cat 1 gene was found in all six resistance genes in the current analysis. Various antibiotic resistance genes are present in *Salmonella* on mobile genetic components, facilitating rapid spread of resistance characteristics to other serotypes or even bacteria of different genera. In line with this, Ahmed and Shimamoto (2012) showed that only tetA gene was found in *S. Typhimurium* [44] out of the six examined genes (*tetA, tetB, tetC, tetD, tetE*, and tetG). Additionally, studies by Adesiji et al. (2014) showed that *tetA* was the most prevalent (100%) [45]. TetA is predominantly present in non-typhoidal *Salmonella*, highlighting the contribution of the gene to tetracycline resistance. Sul1 and sul2 genes code for sulfonamide resistance. Sul1 gene was found in 82.35% of isolates while sul2 gene was only found in 8.82% of isolates. The predominance of sul1 gene in S. Typhimurium was described by Randall et al. (2004) [46]. Ahmed et al. (2016) also discovered sul1 gene in *Salmonella* species resistant to 96.7% of sulfonamides.

Different prevalences of resistance genes were noted; the most prevalent gene among poultry strains was floR. The results of this analysis show that S. typhimurium is the most common serotype. Most antimicrobial drugs under investigation are not effective against poultry *Salmonella* isolates, posing a public health problem. This problem can be exacerbated by the continued use of drugs in chicken farming. Therefore, prudent use of antibiotics appears necessary to prevent the formation of resistant Salmonella strains.

The results of this study provide current data on the frequency of *S. typhimurium* in poultry meat sold in retail establishments in the Iranian city of Shahrekord. These *Salmonella* typhimurium isolates recovered from poultry meat exhibited serovar-specific pathogenicity genes as well as various important resistance genes. It is crucial to keep in mind that control is necessary at every stage of the food chain.

## Data Availability

The datasets used and analyzed during the current study are available from the corresponding author upon reasonable request.
